# Acidic Stress Triggers Sodium-Coupled Bicarbonate Transport and Promotes Survival in A375 Human Melanoma Cells

**DOI:** 10.1038/s41598-019-43262-y

**Published:** 2019-05-02

**Authors:** Oscar C. Y. Yang, Shih-Hurng Loh

**Affiliations:** 1 Division of Structural Biology, Wellcome Trust Centre for Human Genetics, University of Oxford, Roosevelt Drive, Oxford, OX3 7BN United Kingdom; 2 Department of Pharmacology, National Defense Medical Center, Taipei, Taiwan; 3 Department of Pharmacy Practice, Tri-Service General Hospital, National Defense Medical Center, Taipei, Taiwan

**Keywords:** Biological sciences, Cancer, Skin cancer, Melanoma

## Abstract

Melanoma cells preserve intracellular pH (pH_i_) within a viable range despite an acidic ambient pH that typically falls below pH 7.0. The molecular mechanisms underlying this form of acidic preservation in melanoma remain poorly understood. Previous studies had demonstrated that proton transporters including the monocarboxylate transporter (MCT), the sodium hydrogen exchanger (NHE), and V-Type ATPase mediate acid extrusion to counter intracellular acidification in melanoma cells. In this report, the expression and function of the Sodium-Coupled Bicarbonate Transporter (NCBT) family of base loaders were further characterized in melanoma cell lines. NCBT family members were found to be expressed in three different melanoma cell lines – A375, MeWo, and HS695T – and included the electrogenic sodium-bicarbonate cotransporter isoforms 1 and 2 (NBCe1 and NBCe2), the electroneutral sodium-bicarbonate cotransporter (NBCn1), and the sodium-dependent chloride-bicarbonate exchanger (NDCBE). These transporters facilitated 4,4′-diisothiocyanatostilbene-2,2′-disulfonic acid (DIDS)-dependent pH_i_ recovery in melanoma cells, in response to intracellular acidification induced by ammonium chloride prepulse. Furthermore, the expression of NCBTs were upregulated via chronic exposure to extracellular acidification. Given the current research interest in the NCBTs as a molecular driver of tumourigenesis, characterising NCBT in melanoma provides impetus for developing novel therapeutic targets for melanoma treatment.

## Introduction

Intracellular acidification represents one of the defining parenchymal features that influence melanoma metabolism^1^. As a consequence of Warburg aerobic glycolysis and upregulated hypoxic transcriptional reprogramming^2^, endogenous metabolic acids accumulate within the melanoma cytosolic space, subjecting melanoma cells to the physiological stress of cytoplasmic acidification^3^. The cutaneous locality of melanoma growth also increases the vulnerability of melanoma to the effects of intracellular acidification from its pH micromilieu^4^. Vertical growth and invasion into the acidic skin surface, often referred to as the “Acid Mantle”^5^, requires melanoma cells encounter a hostile pH environment in the superficial layers of the stratum granulosum, where pH had been recorded to be as low as pH 5.0^6^. Active maintenance of intracellular pH (pH_i_) at above pH 7.2 during periods of acidic stress permits melanoma cells to continue to proliferate and evade apoptosis^7^. In doing so, melanoma cells require a functional set of molecular acid extruders to neutralise and balance its internal pH in response to intracellular acidification^8^.

Major acid extruder protein families that are known to operate in melanomas include the monocarboxylate transporter (MCT), the sodium-hydrogen exchanger (NHE)^9^, and the V-Type ATPase^10^. MCT consists of an enzymatic solute carrier that mediates the equimolar cotransport of monocarboxylates and protons across the plasma membrane^11^. The MCT isoform 1 has been shown to be the predominant isoform expressed in melanoma cells^12^. Since lactate and protons represent the majority of accumulated intracellular acidic metabolites in the glycolytically favoured melanoma cells^4^, MCT1 also constitutes the dominant modality of pH_i_ regulation in melanoma cells^12^. Intracellularly located protons are additionally removed from the cytosol by NHEs in melanomas^9^. NHE enzymatically catalyzes the movement of protons across the plasma membrane via the active coupling to sodium antiport^13^. NHE isoform 1 is predominantly expressed in human melanomas, and amiloride-dependent NHE1 proton transport activity has been detected in cultured melanoma cells^9^. In addition, V-Type ATPase and NHE isoform 3 had been shown to co-localize with melanosomal proteins, and these proton transport effectors regulate melanosomal pH *in vitro*^14^. V-Type ATPase functions as a rotary proton pump that extrudes protons via energy derived from catalytic ATP hydrolysis using its V_1_ subunit^15^. Melanoma pH_i_ is thus tightly controlled by a set of functionally overlapping proton transporters, which regulate intracellular acidity to sustain cellular viability and metabolism in melanoma^16^.

One major acid extruder protein family that has remained poorly characterised to date in melanoma cells is the sodium-coupled bicarbonate transporter (NCBT) family of pH_i_ regulators^4^. Recent studies in breast and pancreatic cancer have identified NCBTs as important molecular drivers of tumourigenesis^17–20^. Members of the NBC transporter family function as Na^+^-coupled ${{\rm{HCO}}}_{3}^{-}$ transporters for the purpose of intracellular ${{\rm{HCO}}}_{3}^{-}$ loading^21^, and these transporters consist of NBCn1 (electroneutral Na^+^-${{\rm{HCO}}}_{3}^{-}$-Cotransporter 1), NBCe1 (electrogenic Na^+^-${{\rm{HCO}}}_{3}^{-}$-Cotransporter 1), NBCe2 (electrogenic Na^+^-${{\rm{HCO}}}_{3}^{-}$-Cotransporter 2), and NDCBE (Sodium-Dependent Chloride-Bicarbonate Exchanger)^22^. Electrogenic NCBTs (NBCe1 and NBCe2) cotransport Na^+^ and ${{\rm{HCO}}}_{3}^{-}$ in a 1:2 or 1:3 stoichiometric ratio^18^. Such transport activity results in the net movement of 1–2 negative charges across the cell membrane per transport cycle^23^. Electrogenic NCBT transport thus carries electrical current and generates membrane potential in addition to its base-loading activity^22^. NBCn1 and NDCBE mediate electroneutral sodium-bicarbonate cotransport^24^, resulting in the completion of a transport cycle without net movement of electrical charge^25^. NDCBE in particular appears to be a hybrid cotransporter/exchanger that cotransports one Na^+^ and two ${{\rm{HCO}}}_{3}^{-}$ into the cell in exchange for a single Cl^−^ ^25^. Together, the NCBTs cooperate to promote tumourigenesis via exerting diverse regulatory effects on pH_i_, bicarbonate, CO_2_, and cellular membrane potential^22^. Therefore, demonstrating the molecular and functional presence of NCBTs in melanoma cells would significantly enhance the understanding of melanoma pathogenesis^26^.

This study used the A375 melanoma cell line model to investigate the effects of extracellular acidification on melanoma biology. Above the extracellular pH (pH_e_) threshold of pH_e_ 6.8, A375 cells remained viable and slowly proliferated. Additionally, A375 cells kept pH_i_ within the viable range via an endergonic reaction that was significantly curtailed upon serum deprivation. This observation prompted a further search for active acid extrusion mechanisms that were responsible for preserving pH_i_ in melanoma cells, and yielded definitive evidence of NCBT’s expression and function in melanoma cells. This study further showed that the expression of NCBTs in A375 melanoma cells could be upregulated by the exposure to chronic acidity, a relevant pathophysiological feature of NCBT’s regulation in melanoma cells.

## Results

### A375 melanoma cells survive and proliferate in mildly acidic pH

**Figure 1:**
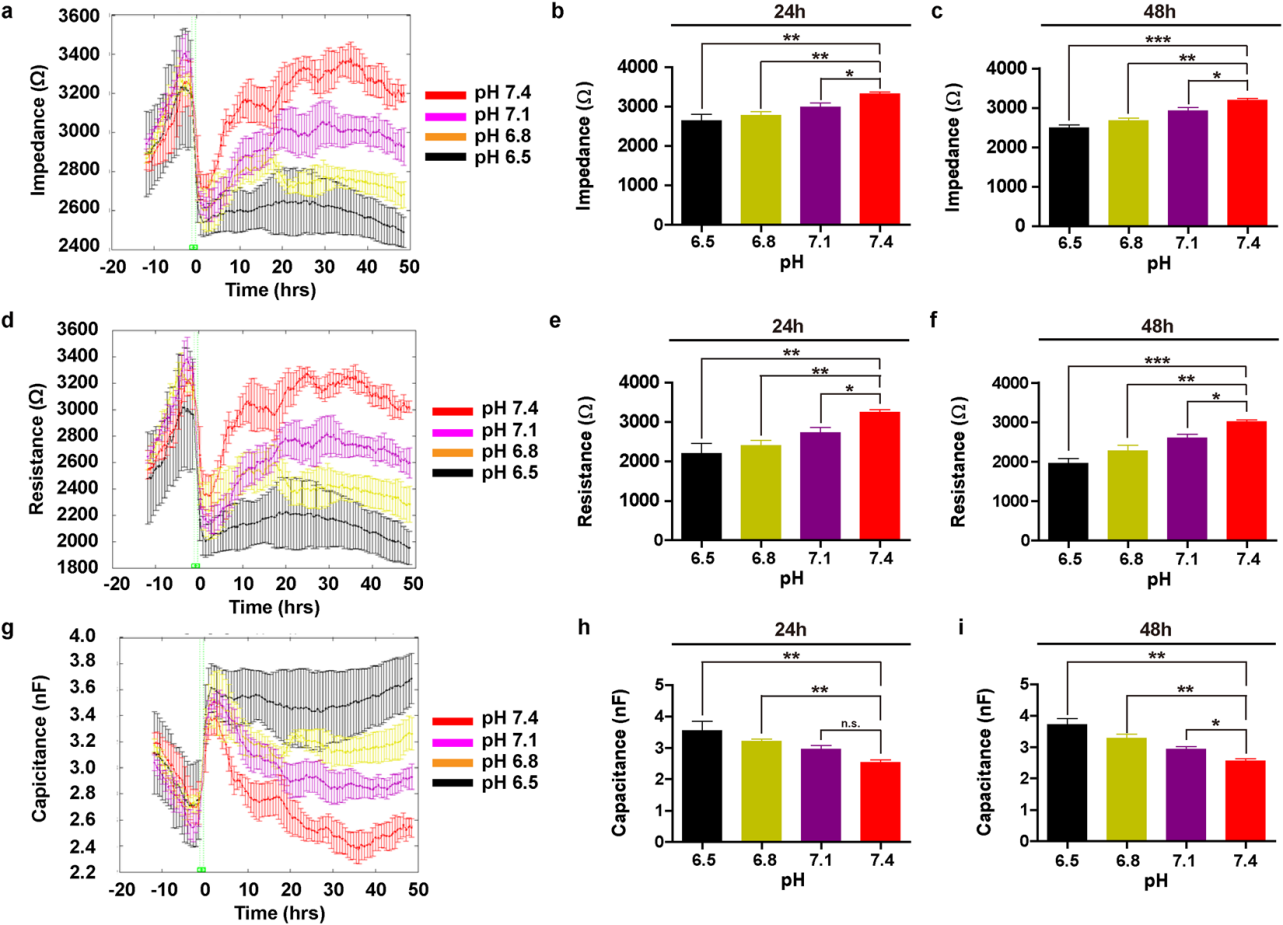
Proliferation Rate of Melanoma Cell Line A375 is Dependent on Ambient pH. (**a**) Impedance measurement of ECIS electrodes continuously recorded for 48 hours (n = 4). A375 cells were seeded into the electrode-fitted wells, and at 0-hour, pH of the culturing media was adjusted to 7.4, 7.1, 6.8, and 6.5, respectively. Quantification of electrode impedance values at (**b**) 24-hours and (**c**) 48-hours is shown (n = 4). (**d**) Resistance and (**g**) Capacitance measurements of ECIS electrodes continuously recorded for 48 hours (n = 4). Quantification of electrode resistance was performed at (**e**) 24-hours and (**f**) 48-hours (n = 4). That of electrode capacitance is shown in (**h**,**g**), respectively (n = 4). Error bars: mean ± SEM. *P < 0.05; **P < 0.01; ***P < 0.001; n.s.: Non-significant. Statistical significance was calculated using unpaired *t*-test.

A375 cells were cultured in different pH conditions and cell proliferation, viability, and intracellular pH were evaluated over 48 hours. As indicated in Fig. 1, acidic pH reduced the proliferative cell coverage rate of detecting microelectrodes in a dose-dependent manner, as measured and quantified by electrode impedance (Fig. 1a–c), resistance (Fig. 1d–f), and capacitance (Fig. 1g–i) at the 24- and 48-hr time points. Values for electrode impedance and resistance were proportional to the rate of proliferative cell coverage of electrodes over time, whereas capacitance was inversely related to electrode coverage (Fig. 1). Cellular electrode coverage occurred at the fastest rate in the neutral pH 7.4 condition, and slowest in the acidic pH 6.5 condition. At 48 hr, absolute cell coverage also plateaued at the highest impedance value in A375 cells cultured in pH 7.4 (Fig. 1a). Absolute cell coverage at 48-hr increased incrementally with the rise in culturing pH in a dose-titrated fashion (Fig. 1a). Interestingly, aside from pH 6.5, where net proliferation was negative (Fig. 1a–c), A375 cells were still able to proliferate slowly at the mildly acidotic threshold of pH 6.8 (Fig. 1a–c).

**Figure 2:**
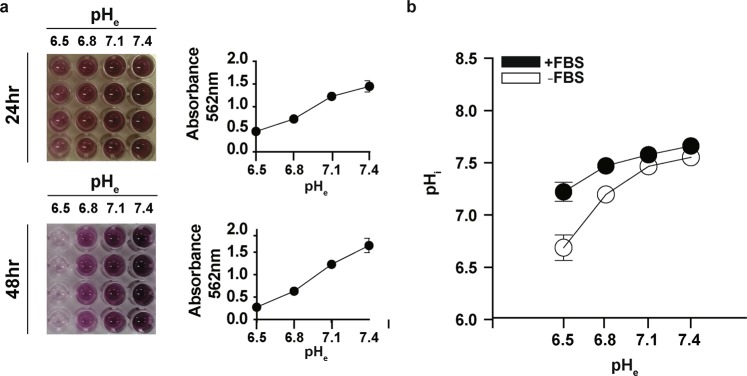
Viability and Intracellular pH of Melanoma Cell Line A375 are Dependent on Ambient pH. (**a**) A375 melanoma cells were plated at low density and incubated in culturing media that had pH adjusted to 7.4, 7.1, 6.8, and 6.5, respectively. At 24- and 48-hour time points, the cells were treated with MTT (n = 4). Quantification of the colourimetric absorbance at 562 nm was recorded for the wells at the prescribed time points as shown (n = 4). (**b**) Quantification of the changes in intracellular pH (pH_i_) after 24-hours of exposure to decreasing extracellular pH (pH_e_). Cells were incubated with or without FBS (n = 4). Error bars: mean ± SEM.

To confirm the above findings, 3-(4,5-dimethylthiazol-2-yl)-2,5-diphenyltetrazolium bromide (MTT) viability assay was performed in A375 cells cultured in same pH acidities over the same 48 hr period (Fig. 2). As shown in Fig. 2a, dose dependent reduction in formazan production was already detectable with decreasing ambient pH conditions at 24-hours, and this effect became more apparent at 48-hours. A375 cells were the most viable at pH 7.4, and least viable at the acidic pH of 6.5 (Fig. 2a). Again, pH 6.8 appeared to be the threshold for cell viability in A375, as formazan formation was virtually undetectable at pH 6.5 at the 48 hr time point (Fig. 2a). pH 6.8 thus represented an appropriate pH to investigate how A375 cells were able to adapt to survive and slowly proliferate in acidic conditions.

### A375 melanoma cells survive acidic pH by preserving intracellular pH neutrality

To determine how A375 cells were able to survive and proliferate at mildly acidic conditions of pH 6.8, BCECF spectrophotometry was used to measure the intracellular pH (pH_i_) in A375 cells cultured for 24 hr in different acidity values (Fig. 2b). Surprisingly, despite lowering the extracellular pH (pH_e_) of the culturing medium to pH_e_ 6.8, A375’s intracellular pH (pH_i_) remained at 7.45 ± 0.05 (n = 4) in the presence of FBS, and at 7.24 ± 0.03 (n = 4) in serum free conditions (Fig. 2b). Such pH_i_ values were still compatible with survival and cell proliferation in A375 cells, which may have at least in part explained their slow proliferation despite acidification of their external environment. In contrast, at the pH_e_ of 6.5, pH_i_ in A375 significantly decreased to 7.27 ± 0.12 (n = 4) in the presence of FBS, and to 6.73 ± 0.23 (n = 4) in serum free conditions (Fig. 2b). Intracellular pH differences in the presence or absence of FBS may be due to changes in metabolic proton production and not efflux (or both), so we considered that at the mildly acidic conditions of pH_e_ 6.8, some form of FBS-dependent, endergonic acid-extrusion mechanism may be in place in A375 cells, and that this mechanism preserved intracellular pH despite the imposed extracellular acidity in A375 cells.

### Multiple proton extruders act synergistically to neutralise intracellular pH in A375 cells in response to acute intracellular acidification

**Figure 3:**
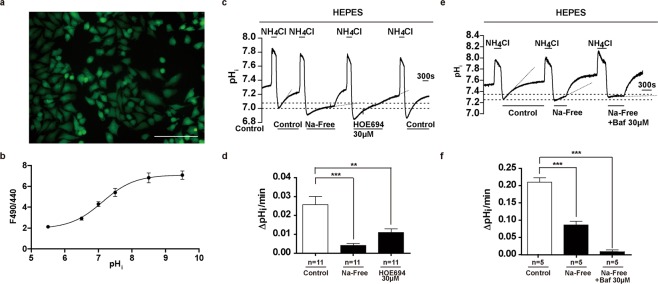
NHE and V-Type ATPase Mediate Acid Extrusion in Melanoma Cell Line A375. (**a**) Fluorescence of intracellular BCECF in A375 cells. (**b**) Quantification of the relationship between F490/440 fluorescence ratio and pH_i_ in A375 cells (n = 9). (**c**) Representative pH_i_ signal of A375 cells serially treated with 20 mM NH_4_Cl prepulses, Sodium-free condition and HOE694 treatment were applied during the pH_i_ recovery periods, as indicated. (**d**) Quantification of pH_i_ recovery rates under control, sodium-free condition, and HOE694 treatment (n = 11). (**e**) Representative pH_i_ signal of A375 cells serially treated with 20 mM NH_4_Cl prepulses, Sodium-free conditions with and without Bafilomycin A1 treatment were applied during the pH_i_ recovery periods, as indicated. (**f**) Quantification of pH_i_ recovery rates under control, sodium-free condition with, and sodium free condition without Bafilomyin A1 (n = 5). Error bars: mean ± SEM. **P < 0.01; ***P < 0.001. Statistical significance was calculated using unpaired *t*-test.

A review of current literature^2,4^ suggested that the proton extruders known to actively regulate intracellular pH in melanoma cells included the sodium hydrogen exchanger (NHE)^9^, the monocarboxylate transporter (MCT)^12^, and the V-type ATPase^14,27^. BCECF microspectrofluorometry was thus used to investigate whether these documented transporters were functional in maintaining pH_i_ balance during periods of acute acidification in A375 cells (Fig. 3). Figure 3a demonstrates the fluorescence of intracellular de-esterified BCECF in A375, under the 488 nm emission filter. Figure 3b shows the calibration and quantification of BCECF fluorescent intensity to pH_i_ values in A375. The pKa of BCECF was determined according to the modified Henderson-Hasselbalch equation^28^ in the form of: pH_i_ = pKa + log_10_ (R − R_min_)/(R_max_ − R), where R represents the F490/440 fluorescence ratio of BCECF at various pH_i_ levels; R_max_ represents the F490/440 fluorescence ratio of the deprotonated form of BCECF (at pH_i_ 9.5); and R_min_ represents the F490/440 fluorescence ratio of the protonated form of BCECF (at pH_i_ 5.5)^28^. pKa of BCECF was determined to be 7.234 for this experiment.

Under fluorescent inverted microscopy with continuous HEPES superfusion of the cells, the NH_4_Cl prepulse technique was used to induce serial acute intracellular acidification events, in order to record and measure the isolated pH_i_ recovery activities of different types of proton transporters (Fig. 3c). Compared to control recovery in the presence of HEPES superfusion (Fig. 3c,d), restricting sodium from the superfusate during the pH_i_ recovery phase significantly reduced the ability of A375 cells to recover from intracellular acidification, as did the application of HOE694, a specific inhibitor of NHE1 (Fig. 3c,d). This experiment showed that upon acute acidic exposure, NHE1 was functionally active in neutralising intracellular pH in A375 cells.

Interestingly, neither sodium-free condition nor HOE694 treatment could completely inhibit pH_i_ recovery in A375 cells, suggesting some form of sodium- and NHE-1 independent mechanism was also active in these melanoma cells (Fig. 3c,d). Concurrent application of bafilomycin A1, the specific V-Type ATPase inhibitor during the sodium-free pH_i_ recovery phase achieved near-complete inhibition of pH_i_ recovery in A375 cells (Fig. 3e,f), suggesting that V-Type ATPase and NHE1 were synergistic in mediating recovery from intracellular acidity in the A375 melanoma cells.

**Figure 4:**
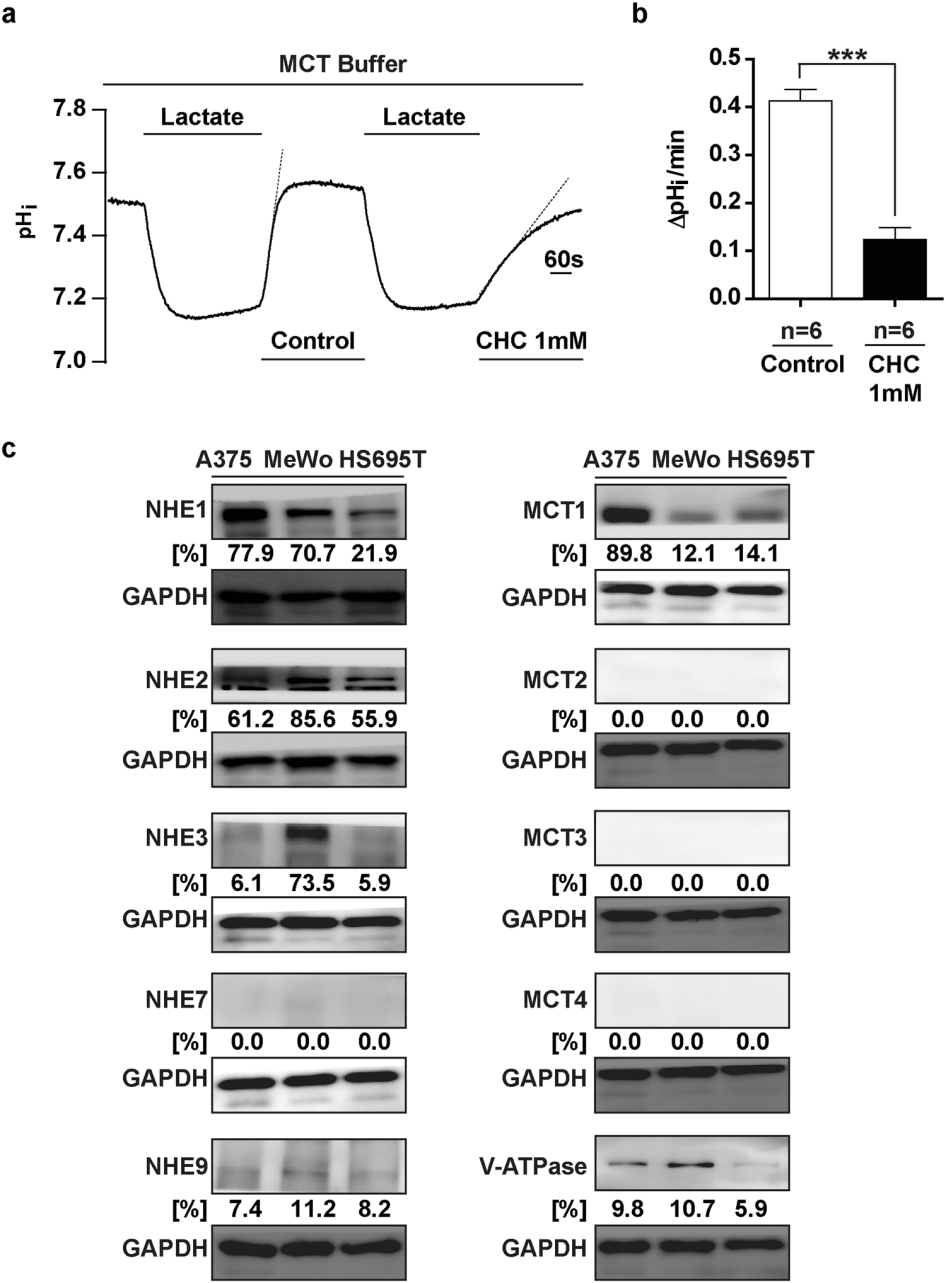
MCT Mediates Acid Extrusion in Melanoma Cell Line A375. (**a**) Representative pH_i_ signal of A375 cells serially superfused with 10 mM sodium lactate. CHC treatment was applied during the pHi recovery period, as indicated. (**b**) Quantification of pH_i_ recovery rates under control and CHC treatments (n = 6). (**c**) Western blots of whole cell lysates from melanoma cell lines A375, MeWo, and HS695T. Primary antibodies: As indicated. Secondary antibodies: HRP conjugated. Loading control: GAPDH. Densitometric quantifications are shown below the Western blots, representing signals normalised to the loading controls, expressed as percentages. Densitometric analysis was performed using the ImageJ software. Error bars: mean ± SEM. ***P < 0.001. Statistical significance was calculated using unpaired *t*-test. Full-length blots are presented in Supplementary Fig. 1.

Next, A375 cells were superfused with sodium lactate, which induced transient acidification of the cytosol (Fig. 4a). Compared to control recovery in the presence of MCT buffer superfusion, CHC treatment – a specific inhibitor of MCT – during the pH_i_ recovery phase also significantly impaired the neutralization of intracellular pH (Fig. 4a,b), suggesting that the MCT transporters were also functionally active in maintaining pH_i_ in A375 cells. These experiments supported the current literature in showing that A375 cells employed a set of functionally overlapping proton extruders to mobilise H^+^ out of the cytosol during periods of acute intracellular acidification^16^.

Western blotting of the whole-cell lysates from melanoma cell lines A375, MeWo, and HS695T showed that different protein isoforms of NHE, MCT, and the V-Type ATPase were indeed expressed in these melanoma cells (Fig. 4c). NHE1 and 2, as well as MCT1 and V-Type ATPase were the dominant isoforms of molecular acid extruders present in the melanoma cell lines surveyed (Fig. 4c).

### Sodium-coupled bicarbonate transporters are expressed and mediate acute pH_i_ recovery in A375 melanoma cells in the presence of ambient CO_2_/bicarbonate

Given that the initial cellular proliferation and viability experiments were performed in the presence of 5% CO_2_ (Figs 1 and 2), with the pH_e_ adjustments achieved by titrating the bicarbonate concentration in the cell culture medium (Figs 1 and 2), it was considered that the above functional and molecular characterisation of the acid extruders in melanoma cells (Figs 3 and 4) – which until now was performed in the absence of CO_2_/Bicarbonate in the superfusate – likely did not account for the entirety of the acid extrusion repertoire present in melanoma cells. Sodium-Coupled Bicarbonate Transporters (NCBT) represents an important class of base loaders also proven to counter intracellular acidification by coupling bicarbonate import with transmembrane sodium cotransport^21,22^. A further search of the literature revealed that the presence of bicarbonate transporters in melanoma cells remained poorly determined^2,4^.

**Figure 5:**
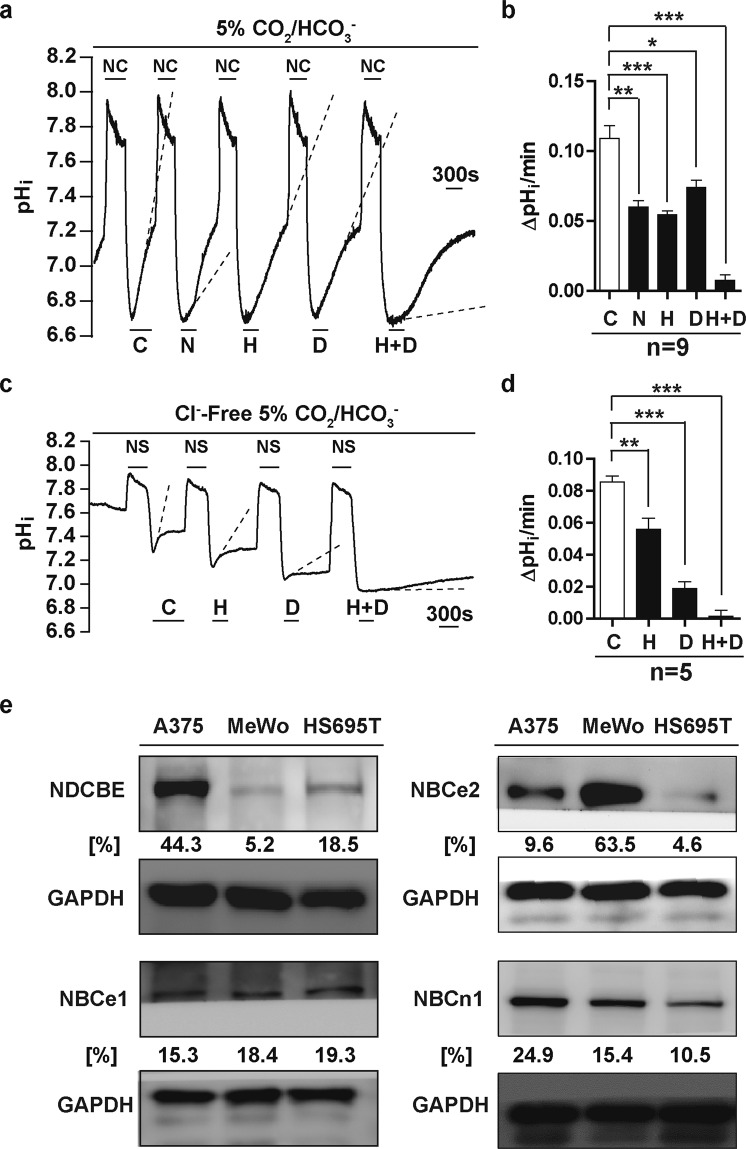
NCBT Mediates Acid Extrusion in Melanoma Cell Line A375. (**a**) Representative pH_i_ signal of A375 cells serially treated with 20 mM NH_4_Cl prepulses (NC) under continuous 5% CO_2_/${{\rm{HCO}}}_{3}^{-}$ solution superfusion. Control (C, equals 5% CO_2_/${{\rm{HCO}}}_{3}^{-}$ solution), Sodium-free superfusate (N), HOE694 30 μM (H), DIDS 30 μM (D), and HOE694 30 μM + DIDS 30 μM (H + D) treatments were applied during the pHi recovery periods, as indicated. (**b**) Quantification of pH_i_ recovery rates under C, N, H, D, and H + D conditions (n = 9). (**c**) Representative pH_i_ signal of A375 cells serially treated with 20 mM (NH_4_)_2_SO_4_ prepulses (NS) under continuous Cl^−^-Free 5% CO_2_/${{\rm{HCO}}}_{3}^{-}$ solution superfusion. Control (C, equals Cl^−^-Free 5% CO_2_/${{\rm{HCO}}}_{3}^{-}$ solution), HOE694 30 μM (H), DIDS 30 μM (D), and HOE694 30 μM + DIDS 30 μM (H + D) treatments were applied during the pHi recovery periods, as indicated. In this protocol cells were bathed in Cl^−^-Free solution for at least 25 min prior to the application of  the first ammonium sulfate prepulse, a protocol sufficient to remove [Cl^−^]_i_ from A375 cells^58^. (**d**) Quantification of pH_i_ recovery rates under C, H, D, and H + D treatments in Cl^−^-Free 5% CO_2_/${{\rm{HCO}}}_{3}^{-}$ condition (n = 5). (**e**) Western blots of whole cell lysates from melanoma cell lines A375, MeWo, and HS695T. Primary antibodies: As indicated. Secondary antibodies: HRP conjugated. Loading control: GAPDH. Densitometric quantification using ImageJ is shown below the Western blots, representing signals normalised to the loading controls, expressed as percentages. Error bars: mean ± SEM. *P < 0.05; **P < 0.01; ***P < 0.001. Statistical significance was calculated using unpaired *t*-test. Full-length blots are presented in Supplementary Fig. 2.

Therefore, to characterise the functional presence of bicarbonate transporters in A375 cells, we examined the pH_i_ recovery activities of A375 cells in response to NH_4_Cl prepulse, this time in the presence of 5% CO_2_/bicarbonate dissolved in the superfusate (Fig. 5a). Compared to control pH_i_ recoveries, sodium restriction and the application of HOE694 during the pH_i_ recovery phases still significantly reduced the ability of A375 cells to recover from intracellular acidification, (Fig. 5a,b), suggesting that NHE transporters were active in the presence of 5% CO_2_/bicarbonate, and served as positive control for the experiment. Next, A375 cells were treated with 4,4′-Diisothiocyano-2,2′-stilbenedisulfonic acid (DIDS), the specific inhibitor of NCBTs. DIDS significantly reduced pH_i_ recovery in A375 cells in the presence of bicarbonate (Fig. 5a,b). Furthermore, the combinational treatment of HOE694 and DIDS together restricted the rate of pHi recovery in A375 cells in an additive manner (Fig. 5a,b). This experiment showed that in the presence of 5% CO_2_/bicarbonate, the NCBTs were functionally active in mediating acute net acid extrusion in response to intracellular acidification in A375 cells, and that these proton transporters likely acted synergistically to maintain pH_i_ homeostasis in the presence of bicarbonate in the microenvironment.

To further discern the functional contributions of different NCBT subtypes to acute pH_i_ recovery in A375 cells, ammonium prepulses with 20 mM (NH_4_)_2_SO_4_ were conducted in Cl^−^-Free 5% CO_2_/${{\rm{HCO}}}_{3}^{-}$ solution, so to remove the Cl^−^-dependent pH_i_-recovery effects of NDCBE, and to functionally isolate the sodium-bicarbonate co-transporter (NBC) component of the NCBT family. As can be seen in Fig. 5c, ${{\rm{HCO}}}_{3}^{-}$-dependent pH_i_ recovery still occurred in the absence of Cl^−^ (as per the control recovery), suggesting that NBC subtypes were functionally active in A375 cells for pH_i_ homeostasis. When 30 μM DIDS was applied to the cells, ${{\rm{HCO}}}_{3}^{-}$-dependent pH_i_ recovery in the absence of Cl^−^ was significantly reduced compared to control, demonstrating the functional inhibition of NBCe1/NBCe2 in A375 cells in the setting of acute ${{\rm{HCO}}}_{3}^{-}$-dependent pH_i_ recovery. The addition of 30 μM HOE694 with 30 μM DIDS almost completely inhibited ${{\rm{HCO}}}_{3}^{-}$-dependent pHi recovery in the absence of Cl^−^, suggesting that at least in the acute setting, the contribution of the DIDS-insensitive NBCn1^29^ to pH_i_ recovery was relatively minor in A375 cells.

Western blotting of whole-cell lysates from melanoma cell lines A375, MeWo, and HS695T showed that four members of the NCBT family of bicarbonate transporters were expressed to varying degrees in the human melanoma cell lines (Fig. 5c), including the electrogenic sodium-bicarbonate cotransporters 1 and 2 (NBCe1 and 2), the electroneutral sodium-bicarbonate cotransporter 1 (NBCn1), and the sodium-dependent chloride/bicarbonate exchanger (NDCBE) (Fig. 5c). We next tested whether these NCBTs could mediate pH_i_ regulation in A375 cells during chronic acidic exposure.

### The expression of sodium-coupled bicarbonate transporters are upregulated during chronic acidic exposure in A375 melanoma cells

**Figure 6:**
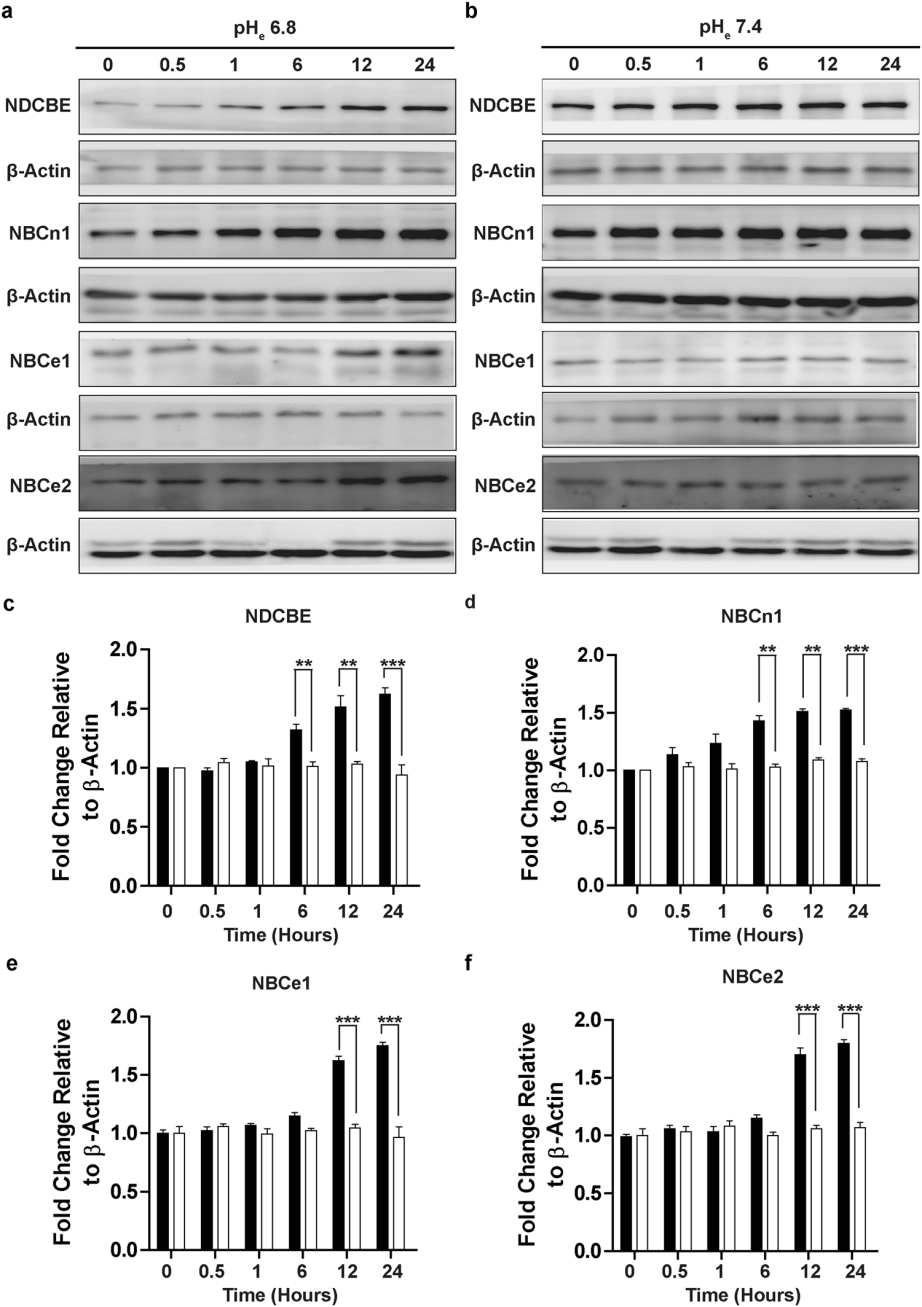
NCBT Protein Expression Levels are Upregulated in Response to Extracellular Acidification in Melanoma Cell Line A375. The protein expression levels of NCBTs in A375 cells exposed to (**a**) pH_e_ 6.8 and (**b**) pH_e_ 7.4 were evaluated over time (hours) by Western blot. Primary antibodies: As indicated. Secondary antibodies: HRP conjugated. Loading control: β-Actin. (**c**–**f**) Densitometric quantifications of the fold changes in the NCBT: β-Actin expression ratios following exposure to pH_e_ 6.8 and 7.4 over time, relative untreated controls at time 0. Densitometric analysis was performed using the ImageJ software. Values were averaged over 4 experiments that were similar to those shown in (**a**,**b**). Error bars: mean ± SEM. **P < 0.01; ***P < 0.001. Statistical significance was calculated using unpaired *t*-test. Full-length blots are presented in Supplementary Fig. 3.

A common feature demonstrated across multiple studies, and shared by individual NCBT members, is their expressional upregulation in response to chronic metabolic acidosis^30–34^. This study next compared the change in protein expression levels of NCBTs over time, when exposed to an extracellular pH of 6.8 or 7.4 (Fig. 6). Exposure to an acidic environment of pH_e_ 6.8 upregulated the expression of the four NCBT transporters – including NBCe1, NBCe2, NDCBE, and NBCn1 – in a time-dependent manner (Fig. 6a), whereas exposure to the control environment of pH_e_ 7.4 exerted minimal effect (Fig. 6b). The NCBTs were measurably upregulated beginning at 6 hours after the exposure to pH_e_ 6.8 (Fig. 6c). Taken together, the results from this study suggest that upon exposure to mild acidic stress, the expression, regulation, and function of different NCBTs in A375 cells were both acutely and chronically potentiated to maintain pH_i_ balance.

## Discussion

This study demonstrated that A375 human melanoma cells exposed to acidic stress relied on functional NCBTs, as well as different modalities of proton transporters to maintain their pH_i_ balance. This study also showed that under acidic stress, the sustenance of pH_i_ within the viable range contributed to cellular proliferation and viability in A375 melanoma cells.

Interestingly, whilst severe acidification has been associated with the induction of acidic cell death across different cell types^35–37^, milder degrees of acidification above pH 6.5 had also been shown to exert protective effects on cell survival^38–40^. In the case of melanoma, survival under mild acidosis contributes to melanomagenesis^3^, through the induction of autophagic and beta-oxidative acidic adaptation^41,42^, with concurrent upregulation of proangiogenic factors VEGF and IL-8^43^. This study showed that the threshold for viability and continued slow proliferation in A375 melanoma cells was pH_e_ 6.8 (Figs 1 and 2). The exact threshold pH_e_ for acidic cell death varies depending on the cell type under acidic exposure^44^; the duration of acidic exposure^30^; and the degree of intracellular acidification^41^. In cortical cultures of neurons for example, the threshold to induce 50% cell death was exposure to pH_e_ 6.4 for 6 hours in the absence of CO_2_/${{\rm{HCO}}}_{3}^{-}$ in the buffering solution^37^. In contrast, even mild acidosis at pH 7.0 induced growth arrest in glioma cells that retained wild type p53 function^45^. Whilst it remains unclear how different absolute thresholds for acidic cell survival are determined in different cell types^41^, in the case of melanoma cells, various proton transport modalities had been shown to contribute to facilitating survival and the upkeep of pH_i_ under acidic stress^3^. There is impetus therefore to individually define the functional presence of proton transporter modalities in melanoma^12^, as carried out in this study, in order to better understand how melanoma cells maintain the pH of its internal environment in response to acidification.

This study provided evidence to show that NCBTs were present and functional in melanoma cells, contributing to pH_i_ maintenance in response to acute and chronic cellular acidification. Many cancer types including breast^19^, pancreatic^46^, and renal cell carcinoma^47^ are known to rely on bicarbonate transporters for pH_i_ regulation, and the melanoma cells in this study were also dependent on the NCBT family. NCBTs import bicarbonate via the coupling to sodium symport, and as such require the active generation of a sodium electrochemical gradient in order to facilitate bicarbonate transport activity^22^. In our hands, DIDS-dependent pH_i_ recovery in 5% CO_2_/${{\rm{HCO}}}_{3}^{-}$ was comparable to the rate of HOE694-dependent pH_i_ recovery, suggesting that NCBTs were an important class of proton transporters that mediated pH_i_ regulation in melanoma cells (Fig. 5a,b). However, CHC-dependent pH_i_ recovery from lactate withdrawal was still at least 2 orders of magnitude faster than either NHE or NCBT in the A375 cells (Fig. 4b), suggesting that MCTs were the most dominant form of acid extrusive mechanism in melanoma cells, consistent with previous experiments by Wahl and Colleagues^12^.

This study further dissected the relative functional contributions of NBC subtypes, in mediating acute ${{\rm{HCO}}}_{3}^{-}$-dependent pH_i_ recovery in A375 cells. In the setting of Cl^−^-free superfusion, the functional presence of NBCe1/2 was demonstrated upon DIDS inhibition (Fig. 5c,d). Recent studies had shed light on the importance of electrogenic NBCs in facilitating hypoxia-induced bicarbonate transport and tumour growth in a range of cancer cell lines^48^. Migration in colon and breast cancer cells had also been shown to dependent on NBCe1^49^. This study showed that electrogenic NBCs were expressed and functionally active in A375 cells for carrying out DIDS-sensitive pH_i_ regulation. More experiments are thus needed to investigate the importance of NBCs in facilitating melanomagenesis and melanoma pH_i_ regulation.

This study hypothesised that exposure of melanoma cells to chronic acidic stress would stimulate the expression of the NCBTs. This hypothesis was tested in A375 cells, a melanoma cell line that demonstrated functional NCBT activity and expression. The findings from this study suggested that acidic stress induced by exposure to pH_e_ 6.8 upregulated the expression of four NCBTs in melanoma cells, including NBCn1, NBCe1, NBCe2, and NDCBE. Statistically significant upregulation of NCBTs was measurable beginning at 6 hrs (Fig. 6c). In previous reports, cardiac and renal NBCe1 expression levels had been shown to be upregulated in neonatal mice and rats, respectively, following chronic exposure to metabolic acidosis, secondary to 12% CO_2_ hypercapnia^31,50^. In addition, protein expression of NDCBE in the CA3 region of rat brain hippocampus also increased by 2.5 fold, secondary to the induction of chronic metabolic acidosis^32^. Furthermore, NBCn1 from primary hippocampal neurons had been shown to increase in protein abundance following the lowering of cell culture pH_e_ to 6.8^30^, and in response to chronic metabolic acidosis induced by orally feeding 0.4 M NH_4_Cl to rats^51^. Increased NBCn1 expression was also reported in rodent kidneys following oral administration of NH_4_Cl^33^, HCl^52^, and after induction of hyperkalemic acidosis^53^. The findings from this study are therefore in support of results from other groups, suggesting that in the context of melanoma, NCBT expression may also be regulated by exposure to chronic acidic stress.

Previous reports had shown that MCT, NHE, and V-Type ATPase play functional roles in pH_i_ regulation in melanoma cells^9,12,14^. This study also demonstrated the functional activities of these transporters in A375 cells. Concomitant activity of functionally overlapping acid extruders, along with the NCBTs in A375 suggests that targeted therapeutic strategies that cater towards the existence of multiple functionally redundant proton transport systems may be more effective at lowering tumour pH_i_ than monotherapeutic approaches alone^16^. The presence of NCBTs in melanoma therefore provides further impetus to evaluate these acid extruders as molecular therapeutic targets for melanoma treatment^19,20^.

Changes in pH_i_ and its regulation have been described in melanoma^2,4^. Proton transport exerts influence on acidic cell survival and proliferation^3^. In the case of melanomagenesis, where malignant vertical growth into the acidic skin surface exposes melanoma cells to the extremes of low pH_e_^5,6^, melanoma cells adapt by developing mechanisms for intracellular proton extrusion^54^. Demonstrating sodium-coupled bicarbonate transport in melanoma cells contributes to a greater understanding of melanoma survival under acidic stress, and the identification of novel pH_i_ regulatory mechanisms melanoma cells may provide viable therapeutic targets for furture melanoma treatments^55^.

## Materials and Methods

### Cell culture and reagents

Cell lines A375, HS696T, and MeWo (Bioresource Collection and Research Centre, Taipei, Taiwan) were grown in Dulbecco’s modified Eagle’s medium (DMEM) (+2% L-glutamine), 10% fetal bovine serum (FBS), and 1% penicillin/streptomycin supplement (all from Invitrogen Life Technologies, Carlsbad, CA). For serum-free adjustment of the culturing media, FBS was eliminated from culture. For pH adjustments of the culturing media, DMEM media without sodium bicarbonate (NaHCO_3_) was manually titrated with standardized amounts of NaHCO_3_ to derive the buffered extracellular pH (pH_e_). Osmolarity of the culturing media was balanced using NaCl throughout.

### Electric cell-substrate impedance sensing

Electrical impedance, resistance, and capacitance of A375 cells were measured using the Electric Cell-Substrate Impedance Sensing (ECIS) system (Applied Biophysics, Troy, NY). Cells were seeded in electrode-fitted wells at a sub-confluent density of 30,000 cells per 0.8 cm^2^, and electrode parameters were monitored at 32000 Hz over time. Medium change with different pH_e_ values occurred at 24 hrs after cell seeding.

### 4.3. 3-(4,5-dimethylthiazol-2-yl)-2,5-diphenyltetrazolium bromide (MTT) assay

Cells were seeded in 96-well plates and allowed to attach overnight. Culturing media with different pH_e_ values were administrated to a final volume of 200 μl. After treatment for 24 or 48 hours, the cells were incubated at 37 °C with 20 μl of 3-(4,5-dimethylthiazol-2-yl)-2,5-diphenyltetrazolium bromide (MTT) solution (5 mg/ml, Abcam, Cambridge, UK) for 4 hours. The MTT formazan crystals were dissolved in 150 μl Dimethyl Sulfoxide (Sigma), and a microplate reader (Tecan, Mannedorf, Switzerland) was used to measure colourimetric absorbance at 562 nm.

### BCECF microspectrofluorometry

For cytosolic pH (pH_i_) measurement, cells were loaded with 3 μM BCECF-AM^56^ (Thermo Fisher, Waltham, MA). BCECF epifluorescence was collected at 530 nm with a converted inverted microscope, with alternative and repetitive excitation of the BCECF fluorophore at 490 and 440 nm under monochromator control (Cairn Research, Kent, UK). Signals were digitized using a CED digitizer, and the fluorescence emission ratios were calculated and converted to pH_i_ values by dividing the F490 by the F440 emission. BCECF fluorescence ratio was calibrated using the high-[K^+^] nigericin technique^56^. Few poorly loaded cells showing low signal-to-noise ratio were excluded from analysis. Intracellular acidification was induced by transiently superfusing melanoma cells with 20 mM ammonium chloride (NH_4_Cl, a procedure known as NH_4_Cl prepulse^57^). During Chloride-free treatment, intracellular acidification was alternatively induced by transiently superfusing melanoma cells with 20 mM ammonium sulfate, (NH_4_)_2_SO_4_^58^. Measurement of pH_i_ and recovery rates typically commenced 1 min after NH_4_Cl and (NH_4_)_2_SO_4_ removal, and pH recovery rates were calculated from the change in pH_i_ over a 1-minute time period (d*pH*_*i*_/d*t*).

### Solutions and chemicals

HEPES-Buffered Tyrode’s Solution contained (mM): NaCl 140, KCl 4.5, MgCl_2_ 1, CaCl_2_ 2.5, HEPES 20 and glucose 11. This was adjusted to pH 7.4 at 37 °C with 1 M NaOH.

#### MCT Buffer Solution contained (mM)

NaCl 140, KCl 5, MgCl_2_ 1.2, CaCl_2_ 2, Na_2_HPO_4_ 1, and HEPES 10. This was adjusted to pH 7.4 at 25 °C with 1 M NaOH.

#### 5% CO_2_/${HC}{{O}}_{3}^{-}$ Buffer Solution contained (mM)

NaCl 117, KCl 4.5, MgCl_2_ 1, CaCl_2_ 2.5, NaHCO_3_ 23, and glucose 11. This was adjusted to pH 7.4 at 37 °C with 1 M NaOH, and equilibrated with 5% CO_2_.

#### Cl^−^-Free 5% CO_2_/${HC}{{O}}_{3}^{-}$ Buffer Solution contained (mM)

Sodium Gluconate 120, Potassium Gluconate 4.5, Magnesium Gluconate 1, Calcium Gluconate 1, NaHCO_3_ 23, and glucose 11. This was adjusted to pH 7.4 at 37 °C with 1 M NaOH, and equilibrated with 5% CO_2_.

#### Nigericin Calibration Solution contained (mM)

KCl 140, MgCl_2_ 1, and nigericin 0.01, buffered with one of the following organic buffers: 20 mM MES (pH 5.5 and 6.5), 20 mM HEPES (pH 7.0, 7.5, and 8.5) or 20 mM CAPSO (pH 9.5), and were adjusted to the correct pH with 1 M NaOH at 37 °C.

HOE694 (3-methylsulphonyl-4-piperidinobenzoyl, guanidine hydrochloride, Sanofi-Aventis, Paris, France), Bafilomycin A1 (LC Laboratories, Woburn, MA), CHC (2-Cyano-3-(4-hydroxyphenyl)-2-propenoic acid, Tocris Bioscience, Minneapolis, MN), DIDS (4,4′-Diisothiocyanatostilbene-2,2′-disulfonic acid, Sigma-Aldrich, St. Louis, MO), Lactate (Sigma) were added to the solutions at the indicated concentrations shortly prior to use.

### Antibodies

The following antibodies were used in the experiments: Anti-NBCe1 and Anti-NBCe2 (Origene, Rockville, MD), Anti-NBCn1 (Abgent, San Diego, CA), Anti-NDCBE (Aviva Systems Biology, San Diego, CA). Anti-NHE1 and Anti-NHE2 (Origene), Anti-NHE3 (Thermo Fisher), Anti-NHE7 (GeneTex, Irvine, CA), and Anti-NHE9 (Abcam, Cambridge, UK). Anti-MCT1 (Proteintech, Rosemont, IL), Anti-MCT2 (Bioss, Woburn, MA), Anti-MCT3 (Aviva Systems Biology), and Anti-MCT4 (Proteintech). Anti-V-Type ATPase (Clone H-5; Santa Cruz Biotochnology, CA).

### Western blotting

For SDS-PAGE electrophoresis, denatured proteins were homogenized in sample buffer (Bio-Rad, Hercules, CA), fractionated on Fastcast gels of either 7.5%, 10%, or 12%, depending on the molecular weight of the proteins of interest (Bio-Rad). Fractionated proteins were wet-transferred to PVDF membranes (GE Healthcare, Pittsburgh, PA), blocked in 5% BSA (Bioshop, Burlington, Canada), and probed with primary antibodies (as listed above at the stated dilutions) overnight at 4 °C. Membranes were washed three times in TBST (Sodium Chloride, Trizma Base, 0.1% Tween-20, all from Sigma), and incubated with HRP-conjugated secondary goat anti-rabbit (1:2000, Cell Signaling Technology), or HRP-conjugated secondary horse anti-mouse (1:2000, Cell Signaling Technology) antibodies. Following secondary antibody incubation, the membranes were further washed three times in TBST, and incubated with enhanced chemiluminescence substrate (Bio-Rad). Western blot images were obtained on a UVP BioSpectrum 500 imager (UVP, Upland, CA). Equal loadings were confirmed by probing with rabbit anti-GAPDH antibody (1:10000, GeneTex), or mouse anti-β-actin antibody (1:4000, Genetex). Protein expression levels were quantified by using ImageJ software analysis.

### Statistical analysis

Data were analysed using Prism (GraphPad Software, La Jolia, CA), with the tests specified in the figure legends. Statistical significance was set at *p < 0.05, **p < 0.01, and ***p < 0.001. Data were described as mean ± standard error of the mean (SEM).

## Supplementary information

Dataset 1

Dataset 2

## Data Availability

The datasets generated during and/or analysed during the current study are available from the corresponding author on reasonable request.
