# Supplementary material for: Acidic Stress Triggers Sodium-Coupled Bicarbonate Transport and Promotes Survival in A375 Human Melanoma Cells
Source: Sci Rep. 2019 May 2;9:6858. doi: 10.1038/s41598-019-43262-y (PMC6497716; doi:10.1038/s41598-019-43262-y)
Supplement: Supplementary file 2 — Dataset 2 [file 41598_2019_43262_MOESM2_ESM.pdf]

Title page

Title: Acidic Stress Triggers Sodium-Coupled Bicarbonate Transport and Promotes Survival in A375 Human Melanoma Cells

Authors: Oscar C.Y. Yang [1,2] and Shih-Hurng Loh [1,2,3]*

*

Authors' affiliations:

[1] **Division of Structural Biology, Wellcome Trust Centre for Human Genetics, University of Oxford, Roosevelt Drive, Oxford OX3 7BN, United Kingdom**.

[2] **Department of Pharmacology, National Defense Medical Center, Taipei, Taiwan**.

[3] **Department of Pharmacy Practice, Tri-Service General Hospital, National Defense Medical Center, Taipei, Taiwan**.

***** Correspondence: shloh@mail.ndmctsgh.edu.tw

Shih-Hurng Loh,

Department of Pharmacology, National Defense Medical Center, Taipei, Taiwan.

No.161, Sec. 6, Minquan E. Rd., Neihu Dist., Taipei City 11490, Taiwan (R.O.C.)

Phone: +886-02-87923100

E-mail: shloh@mail.ndmctsgh.edu.tw

Keywords: Melanoma; Proton Transport; Intracellular Acidification; Sodium-Coupled Bicarbonate Transport; NCBT; NBC; NHE; MCT; V-Type ATPase.

Total numbers of Figures: 3

Fig. S1

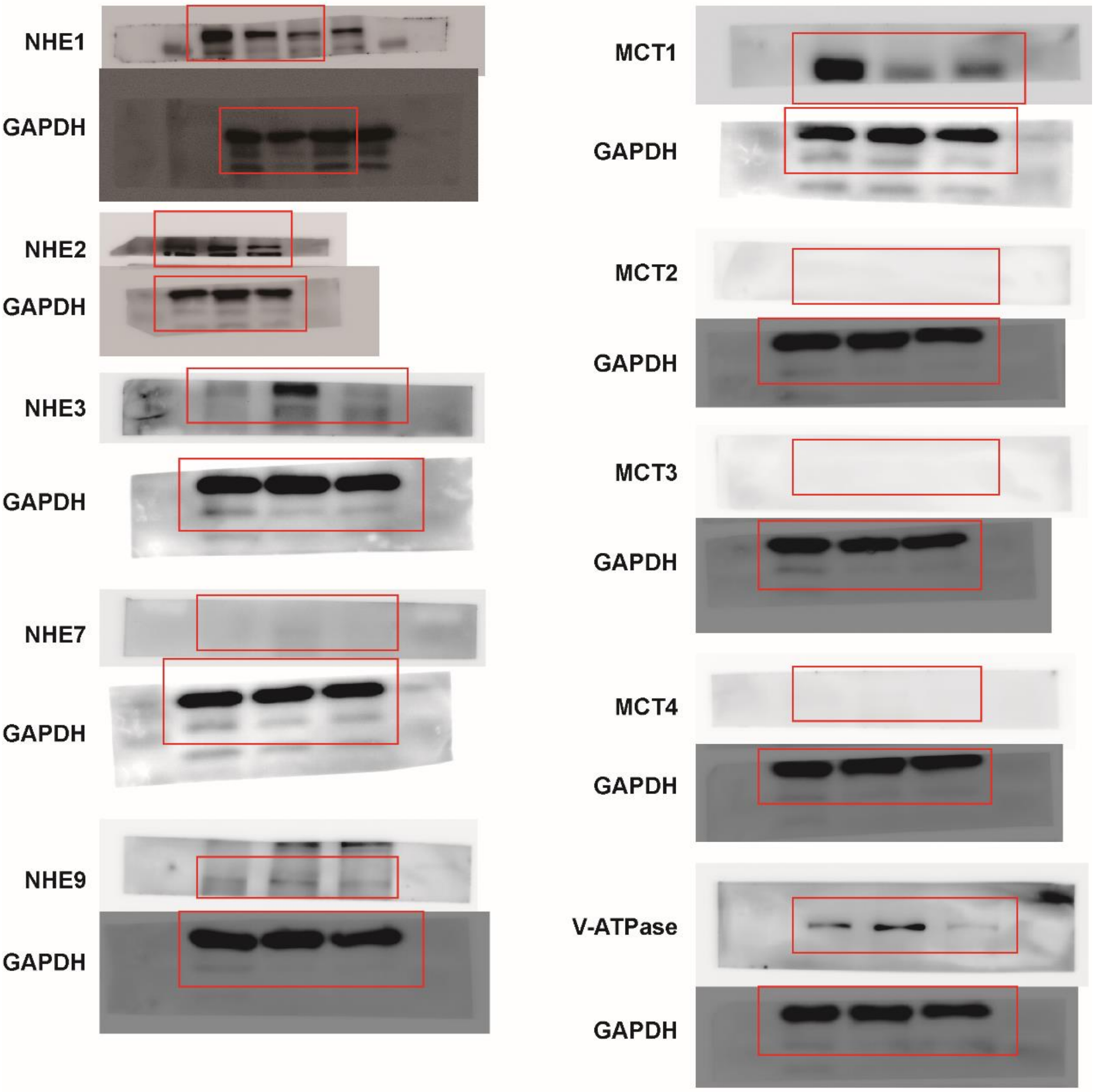

Supplementary Figure 1. Unprocessed scans of original western blots for Figure 4.

Fig. S2

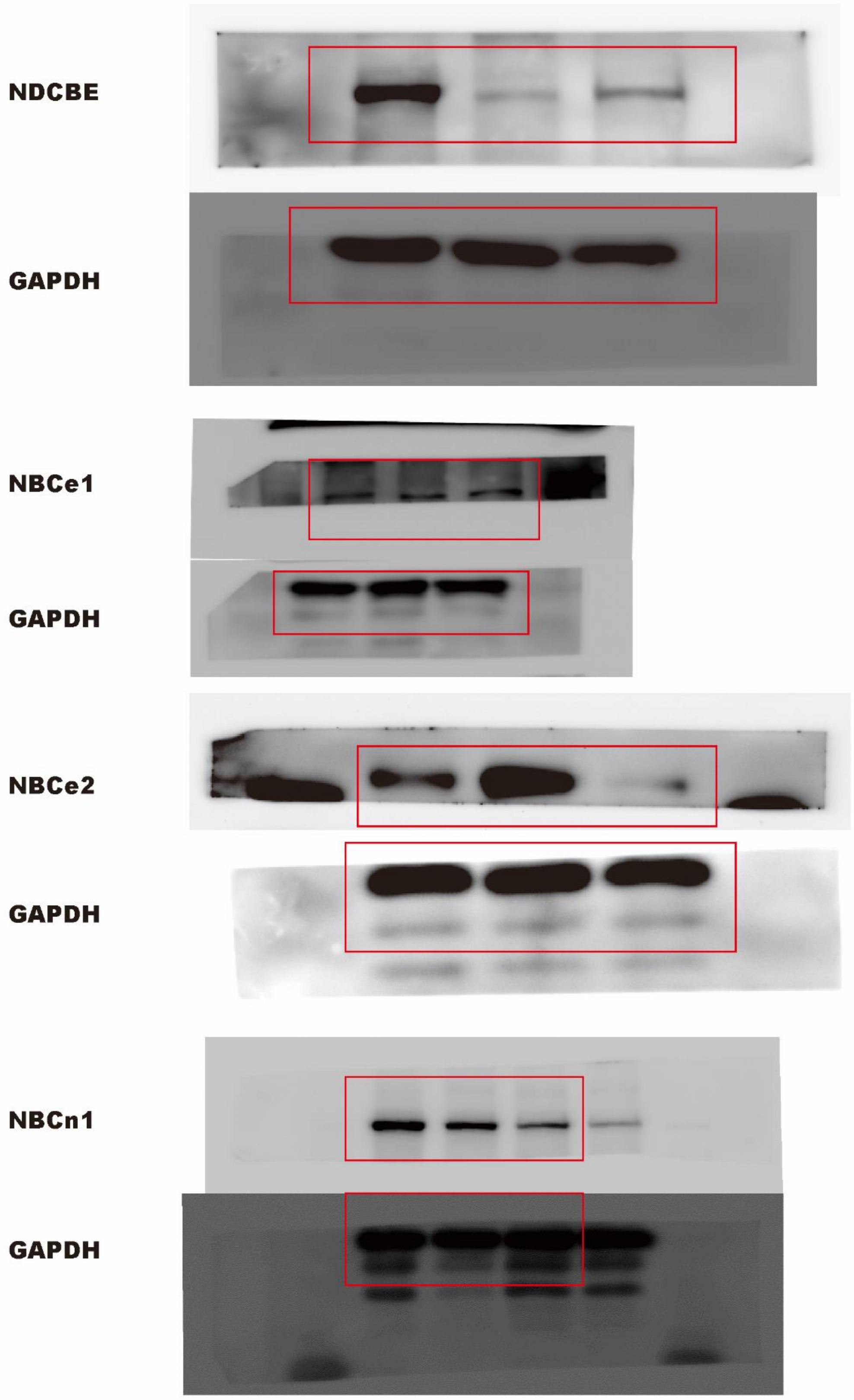

Supplementary Figure 2. Unprocessed scans of original western blots for Figure 5.

Fig. S3

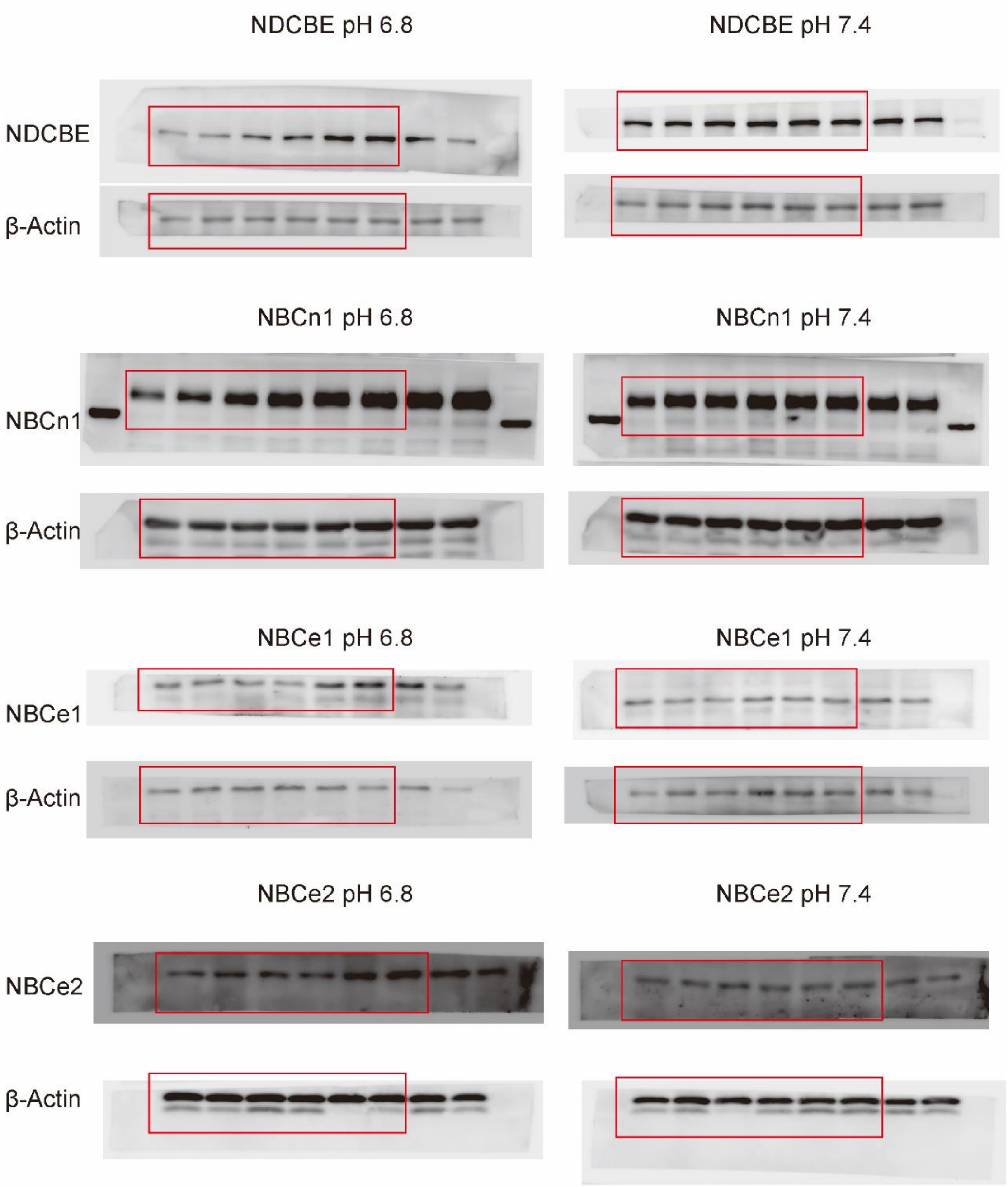

Supplementary Figure 3. Unprocessed scans of original western blots for Figure 6.
